# A human *Myogenin* promoter modified to be highly active in alveolar rhabdomyosarcoma drives an effective suicide gene therapy

**DOI:** 10.1038/s41417-020-00225-0

**Published:** 2020-09-25

**Authors:** Johanna Pruller, Isabella Hofer, Massimo Ganassi, Philipp Heher, Michelle T. Ma, Peter S. Zammit

**Affiliations:** 1 King’s College London, Randall Centre for Cell and Molecular Biophysics, London, SE1 1UL UK; 2 King’s College London, School of Biomedical Engineering and Imaging Sciences, St Thomas’ Hospital, London, SE1 7EH UK

**Keywords:** Gene regulation, Targeted therapies, Sarcoma

## Abstract

Rhabdomyosarcoma is a rare childhood soft tissue cancer whose cells resemble poorly differentiated skeletal muscle, expressing myogenic proteins including MYOGENIN. Alveolar rhabdomyosarcoma (ARMS) accounts for ~40% of cases and is associated with a poorer prognosis than other rhabdomyosarcoma variants, especially if containing the chromosomal translocation generating the PAX3-FOXO1 hybrid transcription factor. Metastasis is commonly present at diagnosis, with a five-year survival rate of <30%, highlighting the need for novel therapeutic approaches. We designed a suicide gene therapy by generating an ARMS-targeted promoter to drive the herpes simplex virus thymidine kinase (HSV-TK) suicide gene. We modified the minimal human *MYOGENIN* promoter by deleting both the NF1 and MEF3 transcription factor binding motifs to produce a promoter that is highly active in ARMS cells. Our bespoke ARMS promoter driving HSV-TK efficiently killed ARMS cells in vitro, but not skeletal myoblasts. Using a xenograft mouse model, we also demonstrated that ARMS promoter-HSV-TK causes apoptosis of ARMS cells in vivo. Importantly, combining our suicide gene therapy with standard chemotherapy agents used in the treatment of rhabdomyosarcoma, reduced the effective drug dose, diminishing deleterious side effects/patient burden. This modified, highly ARMS-specific promoter could provide a new therapy option for this difficult-to-treat cancer.

## Introduction

Rhabdomyosarcoma is the commonest form of childhood soft tissue cancer, affecting 1:150,000 children [1], with an overall favourable prognosis. However, prognosis correlates with classification into two major subtypes: embryonal rhabdomyosarcoma (ERMS, >70% five-year survival rate) and alveolar rhabdomyosarcoma (ARMS, <30% five-year survival rate) [2]. ERMS frequently displays mutations in common oncogenes, such as members of the *RAS* family, *FGFR4*, *PIK3CA* and *CTNNB1*, while such mutations leading to either gain- or loss-of-function are rarely consistently present in ARMS [3].

Currently, treatment for ARMS is predominantly restricted to surgery, together with conventional radiation therapy and chemotherapy. A combination of vincristine, actinomycin and cyclophosphamide (VAC) is the common chemotherapy regime used to treat ARMS in North America. According to both the international classification of paediatric sarcomas and the World Health Organisation classification of skeletal muscle tumours, ARMS is classified as ‘High-Risk Malignant’ [4]. Partially due to the high degree of metastasis at diagnosis, the five-year survival rate remains poor (<30%), highlighting the urgent need for novel therapeutic approaches.

Another factor correlating with low survival is the expression of a novel and ARMS-specific hybrid transcription factor generated through an inframe chromosomal translocation. While a small subset of histologically classified ARMS tumours do not express this hybrid transcription factor, these cases are genetically more aligned with ERMS than fusion-positive ARMS [5]. The DNA binding domain of PAX proteins subfamily III members PAX3 or PAX7 fuses in frame with the transactivation domain of FOXO1, generating highly potent chimeric transcription factors, termed PAX3-FOXO1 (chromosomes 2 and 13) or PAX7-FOXO1 (chromosomes 1 and 13) [4]. PAX3-FOXO1 is associated with a poorer prognosis. PAX3 is essential for embryonic/foetal development of skeletal muscle [6], while PAX7 controls specification/regulation of the resident stem cell pool of postnatal muscle as satellite cells [7, 8]. PAX3 and PAX7 operate with members of the myogenic regulatory factor family (Myf5, MyoD, Myogenin and Myf6/Mrf4) of transcription factors in controlling myogenesis [9]. However, PAX3/7-FOXO1 suppress the transcriptional activity of some MyoD-target genes in muscle stem cells [10]. Interestingly, PAX3-FOXO1 has significantly altered DNA binding properties compared to PAX3, even though the DNA recognition sequences remain identical. PAX3 can bind to an E5 target sequence (adjacent homeodomain ATTA motif and paired GTTCC domain) with higher affinity than PAX3-FOXO1, but even with this lower binding affinity, PAX3-FOXO1 is the more potent transcriptional activator [11].

PAX3/7-FOXO1 target genes are enriched in pathways controlling mesodermal development, neural-related gene expression, and myogenic signalling and differentiation [12]. Specifically, *MYOD* and *MYOGENIN* are upregulated by PAX3/7-FOXO1 [13] and PAX3-FOXO1 interacts directly with the *MYOGENIN* promoter in a MYOD independent way [14]. MYOGENIN is normally tightly controlled during myogenesis, being at negligible levels in proliferating myoblasts, but increasing on cell cycle exit, with peak expression during the fusion phase of the myogenic differentiation program. In contrast, *MYOGENIN* expression in ARMS cells is constitutive, and considered a reliable marker for diagnosis [15, 16], yet is unable to drive myogenic differentiation in such sarcoma cells.

Dysregulated and constitutive expression of *MYOGENIN* in ARMS highlights the *MYOGENIN* promoter as a potential tool to regulate an ARMS-specific suicide gene therapy. A transgene comprising a minimal *MYOGENIN* promoter (−130 to +18 bp) driving a *lacZ* reporter replicates temporal and spatial expression of *MYOGENIN* during embryonic myogenesis in mouse [17, 18]. This minimal *MYOGENIN* promoter transgene contains several well-described and evolutionarily conserved DNA binding motifs, including the TATA box, half a palindromic NF1 site, a MEF2, MEF3 and PBX site, and two E-boxes [19], that control its dynamic regulation. The MEF2 binding motif for example, is required for expression in cells in a subset of somites and the limb buds at embryonic day (E) 11.5 in mouse [17, 18].

Considering differences in expression profile and regulation of the *MYOGENIN* promoter in healthy skeletal muscle compared to ARMS, we hypothesised that a modified *MYOGENIN* promoter could generate a novel ARMS-specific promoter, less active in healthy skeletal muscle. This would allow development of gene therapies driven by such an ARMS-specific promoter. The use of tissue-specific promoters to target cancer cells is not novel, having been demonstrated in multiple cancers, such as the cholecystokinin type A receptor promoter in colorectal cancer [20] or the *HTERT* promoter active in >90% of human cancers [21]. A promoter specific for ARMS, ERMS or rhabdomyosarcoma in general has yet to be described.

An ARMS-specific promoter could drive a suicide gene that delivers an apoptosis-inducing therapy into cancer cells. This strategy using ubiquitously active promoters such as *CMV* has been tested in various cancer types (reviewed in [22]), including ARMS [23], and targets chemotherapy-resistant cell lines efficiently [24]. A commonly used suicide gene therapy is combination of herpes simplex thymidine kinase (HSV-TK) with ganciclovir (GCV). A non-toxic pro-drug capable of penetrating dense tumour, GCV is converted into a cytotoxic drug only through phosphorylation by HSV-TK. Monophosphorylated GCV is then converted to tri-phosphorylated GCV by host cell kinases, creating an adenosine analogue incorporated into DNA during synthesis, causing a delay in S and G2-phase, accompanied by induction of apoptosis [25]. In addition, caspase-8, Chk1 activation [26] and mitochondrial damage [25] occur. HSV-TK/GCV is characterised by high safety, efficacy of mediated cell suicide and an abundant choice of alternatives to GCV with reduced side-effects and increased specificity to cell kinases [27].

By modification of transcription binding motifs, we generated a custom minimal human *MYOGENIN* promoter by deleting the MEF3 and NF1 sites to drive HSV-TK with enhanced specificity for ARMS. Upon GCV treatment, our custom miniMg-∆MEF3/NF1-HSV promoter decreased viability in ARMS cells, but not viability in skeletal muscle cells. In vivo, tumour size was significantly reduced by miniMg-∆MEF3/NF1-HSV in an ARMS xenograft mouse model treated with GCV. In addition, the miniMg-∆MEF3/NF1-HSV promoter effectively targeted tumour cells and lowered chemotherapy dose, and so could be employed in combination with a chemotherapeutic regime. In summary, miniMg-∆MEF3/NF1-HSV is a potential supplement to conventional therapies for this difficult-to-treat cancer.

## Materials and methods

### Mice

Animal work was performed in accordance with British law under the provisions of the Animals (Scientific Procedures) Act 1986, as approved by the Ethical Review Process Committee of King’s College London.

12-week-old immuno-compromised female Swiss Nude mice (Crl:NU(Ico)-Foxn1nu) were purchased from Charles River Laboratories and then housed in ventilated cages to acclimatize for 2 weeks. Mice were assigned randomly to different experimental groups, and no blinding was implemented for data analysis. 5 × 10^5^ RH30 cells expressing HSV-TK under the control of LV-miniMg-Full, LV-miniMg-∆MEF3/NF1 or LV-∆miniMg in 100 µl PBS: Matrigel (50:50) were injected subcutaneously into the right flank. Once tumours were visible (from two months post-injection), tumour size was measured with calipers until size reached 300 mm^3^ (day 0), with GCV administration started 24 h later. Animals then received IP injections of 1 mg GCV/PBS every 24 h for 8 days (day 1 to day 8), and were sacrificed the day of the last injection. After sacrifice, tumours were weighed before being dissected into pieces for later protein and mRNA extraction, as well as imaging. Samples for protein and mRNA extraction were flash frozen in liquid nitrogen and stored at −80 °C. Samples for staining were washed in PBS, fixed in 4% paraformaldehyde (PFA)/PBS for 48 h, passed through 15 and 30% sucrose/PBS before being embedded and frozen in OCT and stored at −80 °C until further processing.

Animal experiments were performed in two separate batches. The first batch showed successful tumour growth in 4/4/2 animals for LV-miniMg-Full/LV-ΔminiMg/LV-miniMg-ΔMEF3/NF1, and the second batch 4/3 animals for LV-ΔminiMg/LV-miniMg-ΔMEF3/NF1 respectively.

### Cell culture

RH30 (CVCL_0041) and RH41 (CVCL_2176) were maintained in DMEM GlutaMax (Gibco, 10566016) with 10% foetal calf serum (FBS) and 1% Pen/Strep (Sigma). C25 and 16U myoblasts were maintained in Promocell skeletal muscle growth medium (Promocell, C-23060) with 15% FBS and 1:1000 Gentamycin (Sigma). All cell lines were maintained in a humidified incubator at 37 °C under 5% CO_2_. Differentiation was induced through a medium change to DMEM GlutaMax, with 1:1000 Insulin and 0.5% FBS. RH30 and RH41 cell lines were obtained from The Institute of Cancer Research, London, 16U from the UMMS Wellstone Centre for FSHD (USA) and C25 from the Institut de Myologie (France). Cell lines were not authenticated in our laboratory, RH30, 16U and C25 tested negative for mycoplasma, RH41 was not tested. Cell lines were cultured continuously for <3 weeks.

### Plasmids

We designed LV-miniMg-Full and LV-miniMg-Full-HSV (with the *MYOGENIN* promoter flanked by XbaI and BamHI sites), which was then manufactured by VectorBuilder. Deletions were introduced through site-directed mutagenesis according to manufacturer’s instructions (ThermoFisher, A14604). Mutant promoters were PCR amplified from LV-miniMg constructs and restriction enzyme sites introduced. The *MYOGENIN* promoter in LV-miniMg-Full-HSV was then exchanged with mutant promoters.

### RNA extraction and RT-qPCR analysis

For whole tumour lysates, 30 mg of tumour tissue was thoroughly homogenized with a TissueRuptor (Qiagen, 9002755) in 700 µl RLT lysis buffer. Cultured cells were lysed directly in 350 µl RLT lysis buffer. mRNA was isolated with the RNeasy kit (Qiagen, 74104) according to manufacturer’s instructions. Reverse transcription was performed with Quantitect Reverse transcription Kit (Qiagen, 205311), SYBR green qPCR was performed (Takyon, UF-NSMT-B0101) on biological replicates [3, 4]. Relative gene expression was normalised to *RPLP0*, and values are represented as 2-∆CT. Primer sequences: *RPLP0*, 5′-TGGTCATCCAGCAGGTGTTCGA-3′(forward) and 5′-ACAGACACTGGCAACATTGCGG-3′ (reverse); *eGFP*, 5′-GAAGCGCGATCACATGGT-3′(forward) and 5′-CCATGCCGAGAGTGATCC-3′(reverse); *mCherry*, 5′-GTGACCGTGACCCAGGAC-3′(forward) and 5′-GCGCAGCTTCACCTTGTAG-3′(reverse); *NF1B*, 5′-CAGGGACTGATGTGGCAAATA-3′(forward) and 5′-CCCTCGATGAAGGATGCATAAA-3′(reverse); *HSV-TK1*, 5′- TACCCGAGCCGATGACTTA-3′(forward) and 5′- CGGTGTTGTGTGGTGTAGAT-3′(reverse); *MYOGENIN*, 5′-CCAGGGGTGCCCAGCGAATG-3′(forward) and 5′-AGCCGTGAGCAGATGATCC-3′(reverse); *MYOMAKER* 5′-AAGATGAAGGAGAAGAAGGG-3′(forward) and 5′-GTAGAAGCTGTGGACATAAG-3′(reverse); *MyHC*, 5′-AGCAGGAGGAGTACAAGAAG-3′(forward) and 5′-CTTTGACCACCTTGGGCTTC-3′(reverse); *P21*, 5′-CCGAAGTCAGTTCCTTGTGG-3′(forward) and 5′-CATGGGTTCTGACGGACAT-3′(reverse); *CCND1*, 5′-GCTGTGCATCTACACCGACA-3′(forward) and 5′-TTGAGCTTGTTCACCAGGAG-3′(reverse); *BAX*, 5′-AGCAAACTGGTGCTCAAGG-3′(forward) and 5′-TCTTGGATCCAGCCCAAC-3′(reverse).

### Immunofluorescence

Samples were fixed with 4% PFA/PBS, permeabilised with 0.05% Triton/PBS and blocked with 5% goat serum/PBS for 60 min. Samples were incubated overnight at 4 °C on a rocker with primary antibody mouse-α-MYOGENIN 1:10 (DSHB, F5D). Next day, samples were washed in PBS for 5 min three times and incubated for 60 min at room temperature with α-mouse 488 1:500 (ThermoFisher, A-11001) diluted in PBS. After further washes, nuclei were counterstained with DAPI 1:1000 and mounted in vectashield (VWR, 101098–042). Samples were viewed and imaged on a Leica AxioVert 200 M.

### TUNEL assay

OCT embedded tumours were acclimatized from −80 °C storage temperature to −21 °C in a cryostat and sectioned at 20 µm. TUNEL assay was performed on cryosections according to manufacturer’s instructions (Abcam, ab66110), nuclei were counterstained with DAPI 1:1000, and mounted in vectashield, samples were imaged on a Leica AxioVert 200 M.

### Luciferase viability assay

Cells were seeded at 1000 cells/well in white 96-well plates (Merck, M0187-32EA). After 24 h, the medium was changed to a medium containing luciferase reagents (Promega, G9711) and GCV sodium salt (SantaCruz, 107910–75–8). Luciferase signal was measured 1 h later, and then every 24 h until 72 h post-medium change. Signal obtained at 24, 48 and 72 h was displayed as fold change to the one-hour timepoint.

### Luciferase apoptosis assay

Cells were seeded at 1000 cells/well in white 96-well plates. After 24 h, the medium was changed to medium containing luciferase reagents (Promega, JA1011) and GCV sodium salt. Luciferase signal was measured repeatedly between 1 and 72 h after the medium change. The presence of apoptosis was considered detectable when the signal increased over background levels and the signal is shown for this timepoint (1 h) and 24 h later.

### Cell counting

Cells were seeded at 5000 (RH30), 1000 (RH41, 16U) or 2000 (C25) cells per well in 24 well plates. After 24 h, the medium was changed to medium containing GCV, and cells were counted using a hematocytometer 24 and 48 h later.

### SiRNA transfection

80,000 RH30 or C25 cells were transfected with 1.5 nM SiRNA against NF1B (Qiagen FlexiTube GeneSolution siRNA, GS4781) or scrambled control SiRNA according to the manufacturer’s instructions, for 24 h. Proliferating RH30 cells were fixed and immunolabelled 48 h post transfection. C25 myoblasts were seeded 24 h post transfection, switched to differentiation medium after an additional 24 h, maintained in differentiation medium for 48 h before fixation and immunolabelling.

### Chemotherapy regime

Vincristine sulfate (V8388-1MG, Sigma), Actinomycin D (A1410-5MG, Sigma) and Cyclophosphamide monohydrate (93813–100MG, Sigma) were used at a ratio of 1.5 mg/m^2^: 0.045 mg/kg: 2200 mg/m^2^, as used for patients >3 years of age [28]. A stock of 96 mM VAC (0.061 mM Vincristine: 0.065 mM Actinomycin D: 96 mM Cyclophosphamide) in PBS was used at 5 fold serial dilutions, starting at a concentration of 9.6 µM (1:10,000) to 0.07 µM VAC (1:1,250,000).

Cells were plated and allowed to attach for 24 h, before being treated with a fresh growth medium containing VAC, GCV and a luciferase substrate (Promega, JA1011) and viability was measured 1 and 24-h post treatment. For counting, cells were plated and allowed to attach for 24 h, then treated with VAC and GCV and counted 24 h later.

### Protein extraction and western blot

Tumour tissue was homogenized with a TissueRuptor in a total of 600 µl RIPA buffer (Sigma, R0278), supplemented with 1:100 phosphatase inhibitor cocktails 2 (Merck, P5726-1ML) and 3 (Merck, 524627–1 ML), and 1:7 protease inhibitors (Merck, 11836170001). The lysate was agitated at 4 °C for 2 h, centrifuged at max speed at 4 °C for 20 min, 4× Laemmli buffer was added to the supernatant and samples were boiled for 5 min at 95 °C. 50 µg protein and 5 µl precision plus protein standards dual colour ladder (BioRad, #161–0374) were loaded in a 4–20 % precast gel (BioRad, #4561094) and run for 1 h at 60 V, and transfer to nitrocellulose membrane was performed at constant 70 V for 1.5 h. The membrane was stained first for BAX protein (polyclonal rabbit-α-BAX, CST, #2772) 1:1000 over night at 4 °C, followed by incubation with α-rabbit-HRP secondary antibody (Sigma, GENA934–1ML) at 1:25,000 for 1 h at room temperature, and development with clarity ECL substrate (BioRad, #1705061). The same membrane was then stained against β-TUBULIN (monoclonal mouse -α-β-TUBULIN, DSHB, E7) 1:4000 over night at 4 °C, followed by incubation with α-mouse-HRP secondary antibody (Sigma, NA931V) for 1 h at room temperature, and development with clarity ECL substrate. Blot was imaged on a ChemiDoc imaging system (BioRad, 17001401).

### Statistical analysis

Statistical analysis was performed using GraphPad Prism 8.0. Experiments were performed with an N of at least 3, with detailed N numbers given with each figure. The variance between groups was compared using a Brown Forsythe test and revealed no significant difference. A comparison between two groups was performed using an unpaired homoscedastic two-tailed student’s *t*-test. A comparison of more than two groups was performed using a one-way ANOVA followed by Dunett’s post test as different groups were compared with the control group. *P* < 0.05 were considered significantly different.

## Results

### Generation of an ARMS-specific promoter by modification of the human *MYOGENIN* promoter

**Fig. 1:**
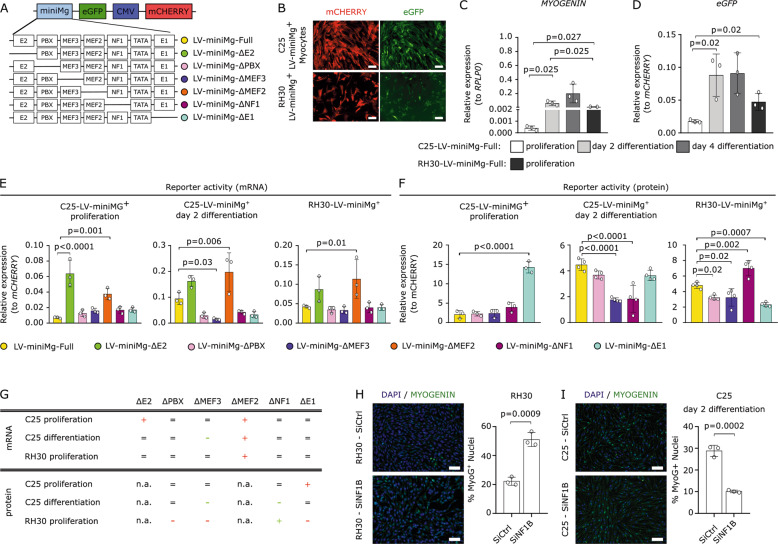
ARMS promoter created by deletion of the MEF3 or NF1 DNA binding site. **a** Schematic of the lentiviral construct where the minimal human *MYOGENIN* promoter (miniMg) drives *eGFP* while *CMV* drives *mCHERRY*. Transcription factor binding motifs in the *MYOGENIN* promoter, together with the deletion constructs, are shown. **b** Representative images of LV-miniMg-transduced C25 myoblasts and RH30 cells. **c**, **d** RT-qPCR for endogenous *MYOGENIN* (**c**) and *eGFP* (**d**) as proxy for minimal *MYOGENIN* promoter activity in C25 myoblasts, myocytes and myotubes, and proliferating RH30 cells. *N* = 3, with significant differences, assessed using a student’s t-test as indicated by bars. **e** RT-qPCR for *eGFP* to measure activity of the various mutant *MYOGENIN* promoters with motif deletion in proliferating and differentiating C25 and proliferating RH30, *N* = 3, statistical difference assessed using a One-Way ANOVA with a Dunett’s post hoc test comparing all samples to control LV-miniMg. **f** Fluorescence measurement of eGFP protein to measure mutant promoter activity in proliferating and differentiating C25 and proliferating RH30, *N* = 3–4, statistical difference assessed using a one-way ANOVA with a Dunett’s post hoc test comparing all samples to control LV-miniMg. **g** Overview of observed changes in promoter activity after motif deletions, in relation to LV-miniMg-Full: ‘+’ indicates a significant increase in promoter activity, ‘−’ a significant decrease, ‘=’ no observed change, ‘n.a.’ is not assessed. Red indicates the exclusion of the promoter from further study, while green indcates inclusion. **h** Immunolabelling for MYOGENIN in RH30 cells after SiRNA-mediated knockdown of *NF1B* (SiNF1B), with quantification of the proportion of MYOGENIN positive nuclei/total nuclei per unit area. **i** Immunolabelling for MYOGENIN in two day differentiated C25 myoblasts after SiRNA-mediated knockdown of *NF1B*, with quantification of the proportion of MYOGENIN positive nuclei/total nuclei per unit area. *N* = 3, statistical significance assessed using a student’s *t*-Test comparing SiNF1B to SiCtrl groups. Data expressed as mean ± SD. Scale bar equals 100 µm.

*MYOGENIN* is constitutively expressed in rhabdomyosarcoma, but only transiently during myogenic differentiation in skeletal muscle, and so was selected for modification to enhance ARMS, but reduce skeletal muscle, expression. A lentiviral construct was generated where the minimal human *MYOGENIN* promoter [17] drives *eGFP*, while the ubiquitously active *CMV* promoter drives *mCHERRY* (termed LV-miniMg-Full—Fig. 1a, b). Evaluation of LV-miniMg-Full was initially performed in the RH30 ARMS cell line [29] and C25 immortalised human skeletal myoblasts [30]. Endogenous *MYOGENIN* expression increased significantly as C25 myoblasts differentiate, with a peak two days after induction of differentiation, as myoblasts fuse and generate immature multinucleated myotubes (Fig. 1c). Conversely, RH30 cells show a moderate constitutive expression of *MYOGENIN* (Fig. 1c). As expected, expression of both *eGFP* and *mCHERRY* from LV-miniMg-Full was robust in C25 myoblasts undergoing myogenic differentiation and in proliferating RH30 cells (Fig. 1b). LV-miniMg-Full activity, measured by *eGFP* normalised to *mCHERRY*, showed the same trend as endogenous *MYOGENIN*, with significantly higher expression of *eGFP* in differentiating C25 myoblasts compared to during proliferation, and moderate levels in RH30 cells. Thus, LV-miniMg-Full mimics *MYOGENIN* expression (Fig. 1d).

To generate a *MYOGENIN* promoter with low/negligible activity in proliferating C25 myoblasts, reduced activity in differentiating C25 myoblasts and stable/increased activity in RH30 cells, we deleted each of six conserved and well-studied transcription factor binding site motifs. Using site-directed mutagenesis, we deleted either the half palindromic NF1, MEF2, MEF3, PBX, E-box E1 or E-box E2 site [19] in LV-miniMg-Full (Fig. 1a). RT-qPCR for *eGFP* expression was used as a proxy to assay mutant *MYOGENIN* promoter activity for each construct in proliferating C25 myoblasts, differentiating C25 myoblasts and RH30 cells (Fig. 1e). Identifying a modification that would cause promoter activity to decrease in proliferating C25 myoblasts compared to LV-miniMg-Full eliminated LV-miniMg-∆E2 and LV-miniMg-∆MEF2. LV-miniMg-∆PBX, LV-miniMg-∆NF1 and LV-miniMg-∆E1 each showed similar activity to LV-miniMg-Full in differentiating C25 myoblasts and RH30 cells, while LV-miniMg-∆MEF3 was significantly reduced in both, and LV-miniMg-∆MEF2 was enhanced in differentiating C25 myoblasts (Fig. 1e).

To confirm promoter activity at the protein level, we also measured eGFP fluorescence normalised to mCHERRY fluorescence (Fig. 1f). The LV-miniMg-∆E1 promoter was excluded due to significantly increased expression in proliferating C25 myoblasts and unchanged activity in differentiating C25 myoblasts. LV-miniMg-∆MEF3 and LV-miniMg-∆NF1 promoters each showed significantly reduced expression in differentiating C25 myoblasts, while the LV-miniMg-∆NF1 promoter had increased activity in RH30 cells. The effect of each mutation is summarised in Fig. 1g.

The reduction of *MYOGENIN* promoter activity after deletion of the MEF3 motif was expected [31], but the role of NF1 in ARMS is little known. *NF1B* is the only NF1 isoform identified as a putative target gene of PAX3-FOXO1 [32]. We performed SiRNA-mediated knockdown of *NF1B* to test if this would affect MYOGENIN expression, to test the trend that we see in our LV-miniMg reporter experiments. Indeed, when *NF1B* was knocked down, there was a significant increase in the proportion of RH30 cells containing MYOGENIN (Fig. 1h), while *NF1B* knockdown significantly reduced the proportion of MYOGENIN positive differentiating C25 myoblasts (Fig. 1i). This confirms that *NF1B* is differently involved in the upstream regulation of MYOGENIN expression in healthy myoblasts and RH30 cells.

Thus, removal of the NF1 or MEF3 DNA binding motif in the minimal human *MYOGENIN* promoter generates promoters with enhanced specificity for RH30 ARMS cells over healthy human C25 myogenic cells, with minimal activity in proliferating C25 myoblasts.

### Custom promoter driving HSV-TK decreases cell viability more in ARMS cells than myogenic cells

**Fig. 2:**
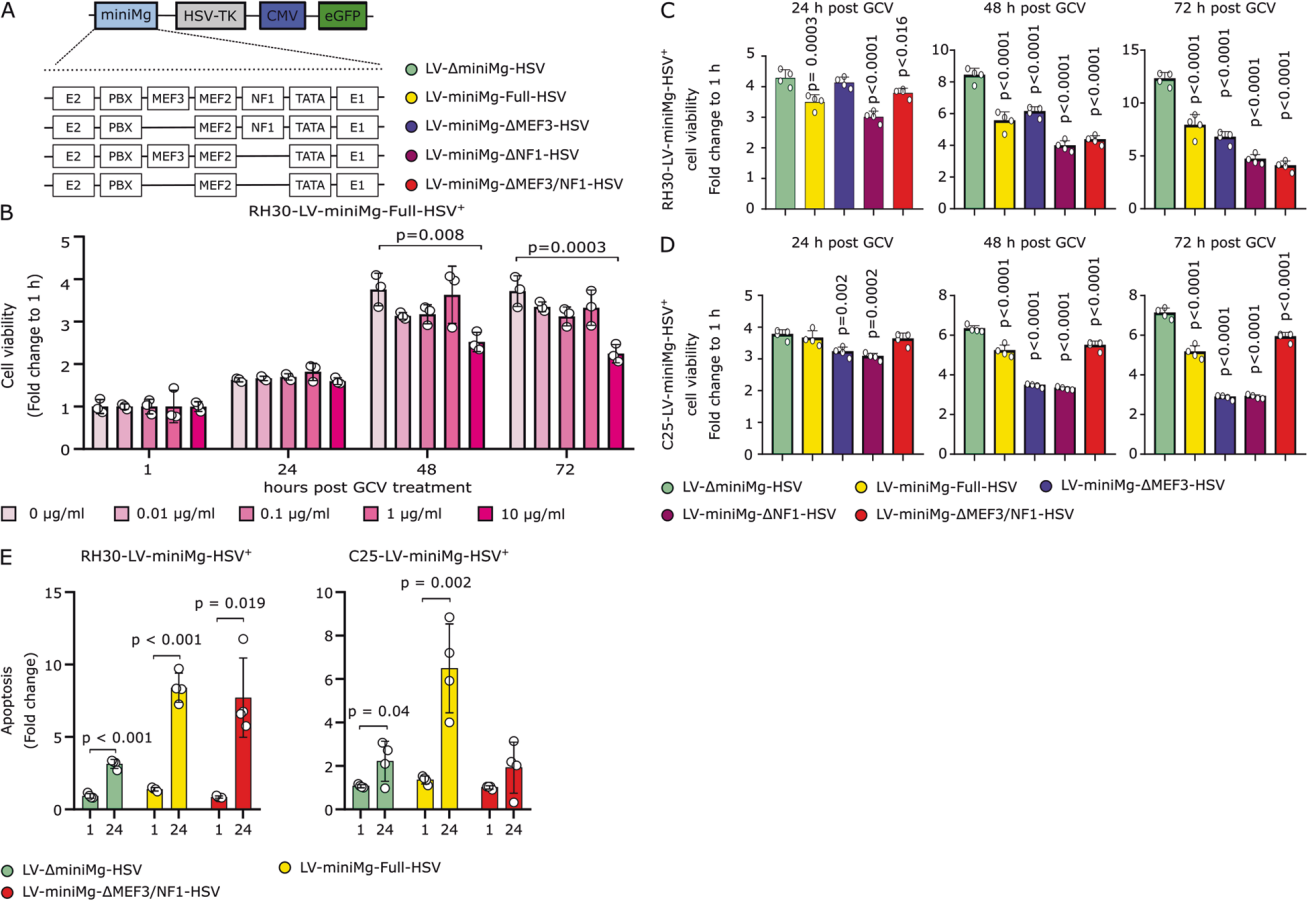
ARMS LV-miniMg-∆MEF3/NF1-HSV promoter reduces RH30 viability through induction of apoptosis. **a** Schematic of the LV-miniMg-HSV lentiviral construct, where the miniMg promoter drives expression of *HSV-TK1* and *CMV* regulates expression of *eGFP*. Variants of the promoter with deletions are depicted below. **b** Concentration of GCV that reduces mean cell viability in RH30 cells stably transduced with LV-miniMg-Full-HSV, *N* *=* 3, statistical difference assessed using a One-Way ANOVA at each timepoint with a Dunnett’s post hoc test, comparing each GCV concentration against control (0 µg/ml). **c** Mean cell viability of RH30 stably transduced with LV-miniMg-Full-HSV, LV-miniMg-∆NF1-HSV, LV-miniMg-∆MEF3-HSV, LV-miniMg-∆MEF3/NF1-HSV or LV-∆miniMg-HSV after 24/48/72 h of GCV treatment. *N* *=* 4, statistical difference assessed using a One-Way ANOVA at each timepoint with a Dunnett’s post hoc test, comparing each promoter with the LV-∆miniMg-HSV control. **d** Cell viability of C25 stably transduced with LV-miniMg-Full-HSV, LV-miniMg-∆NF1-HSV, LV-miniMg-∆MEF3-HSV, LV-miniMg-∆MEF3/NF1-HSV or LV-∆miniMg-HSV after 24/48/72 h of GCV treatment. N *=* 4, statistical difference assessed using a One-Way ANOVA at each timepoint with a Dunnett’s post hoc test, comparing each promoter with the LV-∆miniMg-HSV control. **e** Induction of apoptosis in RH30 and C25 cells stably transduced with LV-∆miniMg-HSV, LV-miniMg-Full-HSV or LV-miniMg-∆MEF3/NF1-HSV and treated with GCV at 1/24 h after apoptosis measurements exceeded background measurements. *N* *=* 4, statistical difference assessed with a student’s *t*-test comparing values at 1 and 24 h after measurements surpassed background values. All data are expressed as mean ± SD.

Since deletion of the NF1 or MEF3 DNA binding domain in the minimal human *MYOGENIN* promoter enhanced specificity for RH30, we also generated a promoter version with both the NF1 and MEF3 sites removed (LV-miniMg-∆MEF3/NF1). To test if those modified human *MYOGENIN* promoters could drive *HSV-TK* expression efficiently in RH30 cells, we generated new constructs with the promoters driving *HSV-TK* in cis with a *CMV* promoter driving *eGFP* (Fig. 2a). We then assessed mean viability of RH30 expressing LV-miniMg-Full-HSV, LV-miniMg-∆NF1-HSV, LV-miniMg-∆MEF3-HSV, LV-miniMg-∆MEF3/NF1-HSV or LV-∆miniMg-HSV (LV backbone lacking a promoter).

To first identify the optimal concentration of GCV, RH30 cells transduced with LV-miniMg-Full-HSV were treated with GCV (10 to 0.01 µg/ml) for 72 h and cell viability measured with a RealTime MT Glo cell viability assay every 24 h (Fig. 2b). This identified 10 µg/ml GCV as the lowest concentration inducing a significant decrease in cell viability.

The effect of all HSV-TK constructs on cell viability was then analysed in parallel using 10 µg/ml GCV for 72 h. Mean cell viability was significantly reduced as early as 24 h after GCV treatment by most promoters except the LV-∆miniMg-HSV control. After 72 h, LV-miniMg-∆MEF3/NF1-HSV was found the most effective at reducing cell viability, suggesting that the deletion of both transcription binding motifs rendered the promoter highly active in RH30 cells (Fig. 2c). In C25 myoblasts, LV-MiniMg-∆MEF3 or LV-MiniMg-∆NF1 significantly decreased viability after 24 h (Fig. 2d). Seventy-two hours after GCV treatment, although myoblast viability was reduced in all groups, LV-MiniMg-∆MEF3/NF1-HSV showed the least effect, showing that deletion of these two DNA binding motifs further reduced promoter activity in skeletal myoblasts.

HSV-TK induces apoptosis in response to GCV, and to confirm that reduced cell viability was indeed due to apoptosis, we quantified Annexin 5 exposed on the cell membrane via a RealTime-Glo™ Annexin V Apoptosis assay. We measured the samples regularly until the observed apoptotic signal increased over background threshold values, and then measured 1 and 24 h after this time-point. LV-miniMg-Full-HSV and LV-miniMg-∆MEF3/NF1-HSV caused a significant increase in apoptosis between 1 and 24 h in RH30 cells. Importantly, LV-miniMg-∆MEF3/NF1 did not change apoptosis in C25 myoblasts, while control LV-miniMg-Full-HSV induced high levels (Fig. 2e). Thus, our ∆MEF3/NF1 *MYOGENIN* promoter is more efficient at inducing apoptosis in RH30 cells than in C25 myoblasts, and can deliver HSV-TK differentially between ARMS and myoblast cells, causing apoptosis in ARMS.

### LV-miniMg-∆MEF3/NF1-HSV driven suicide therapy specifically reduces cell number in multiple ARMS cell lines

**Fig. 3:**
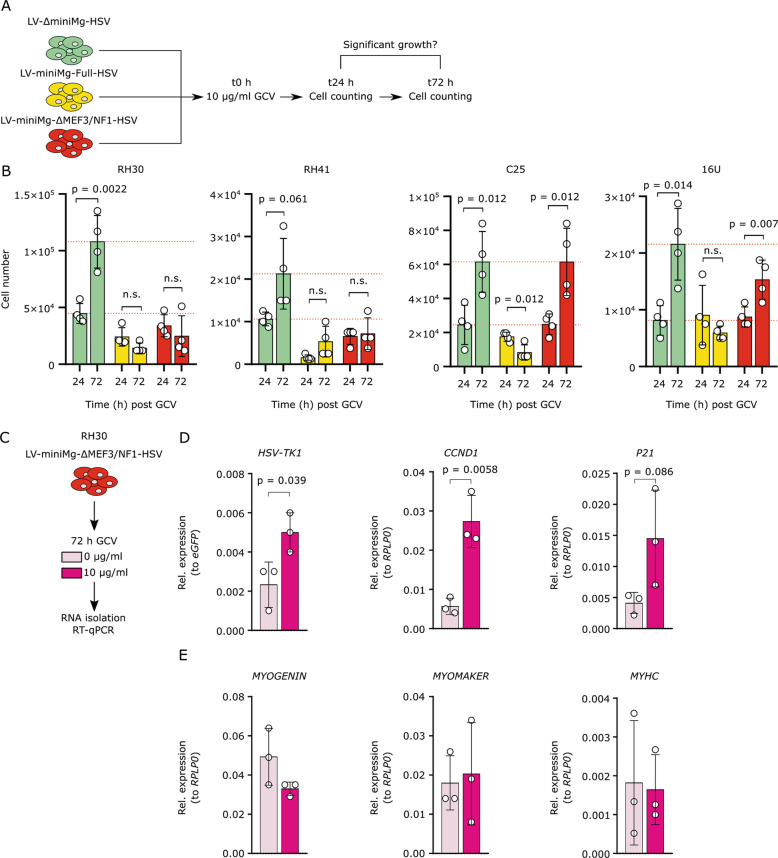
LV-miniMg-∆MEF3/NF1-HSV reduces cell number in ARMS lines but not in human myoblasts. **a** Schematic depicting experimental regime where ARMS (RH30, RH41) and myoblasts (C25, 16U) were transduced with LV-∆miniMg-HSV, LV-miniMg-Full-HSV or LV-miniMg-∆MEF3/NF1-HSV, then treated with 10 µg/ml GCV and counted 24 and 72 h post treatment. **b** Most cell lines (except RH41) increased the mean cell number from 24 to 72 h post-treatment with control LV-∆miniMg-HSV. Cell numbers were unchanged where *HSV-TK* was under control of the miniMgfull-HSV promoter (except C25). Cell numbers were unchanged in RH30 and RH41 lines if the miniMg-∆MEF3/NF1 promoter drove *HSV-TK* expression, while cell numbers increased in both myoblast lines C25 and 16U. Dashed line shows mean cell number of the control group at 24 and 72 h. *N* = 4, statistical significance assessed using a student’s *t*-Test comparing cell numbers between 24 and 72 h. **c** RH30 transduced with the LV-miniMg-∆MEF3/NF1-HSV, were treated with 10 µg/ml GCV or vehicle control and gene expression analysed 72 h later. **d** Mean expression of *HSV-TK*, and cell cycle regulators *CCND1* and *CDKN1A* (encodes P21) and (**e**) expression of myogenesis markers *MYOGENIN*, *MYOMAKER* and *MYHC*. *N* = 3, statistical differences were assessed using a student’s *t*-Test, comparing expression values between vehicle control and samples treated with 10 µg/ml GCV. All data are expressed as mean ± SD.

Having identified LV-miniMg-∆MEF3/NF1-HSV as an effective promoter, we next tested in further cell lines: namely the immortalized RH41 ARMS line [33] and human 16U myoblasts [34], in parallel with RH30 and C25 cells. Lines were transduced with LV-∆miniMg-HSV (negative control), LV-miniMg-Full-HSV (positive control) and LV-miniMg-∆MEF3/NF1-HSV, then incubated with 10 µg/ml GCV and counted 24 and 72 h post-treatment (Fig. 3a). LV-∆miniMg-HSV did not affect cell proliferation between 24 and 72 h in any cell line, while LV-miniMg-Full-HSV induced either no change in cell numbers from 24 to 72 h (RH30, RH41, 16U) or even a significant reduction (C25). Crucially, LV-miniMg-∆MEF3/NF1-HSV differentially affected human cancer cells and myoblasts, with reduced proliferation in the RH30 and RH41 ARMS cell lines (Fig. 3b). However, there were no changes in cell numbers in the C25 and 16U myoblast lines between 24 and 72 h (Fig. 3b). Thus, the introduced modifications in the LV-miniMg-∆MEF3/NF1 promoter reduced toxicity of the suicide gene therapy for human skeletal muscle myoblasts (Fig. 3b).

To directly measure changes in gene expression due to GCV treatment, RH30 cells were transduced with LV-miniMg-∆MEF3/NF1-HSV, treated with either 10 µg/ml GCV or vehicle control, and gene expression quantified by RT-qPCR after 72 h (Fig. 3c). Mean expression of *HSV-TK* remained robust after treatment with GCV, suggesting that an HSV-TK positive population still exists after 72 h and so a longer treatment period could see a further reduction of cell viability (Fig. 3d). There was a significant upregulation of *CCND1* and a trend towards higher *CDKN1A* (encodes P21*)* mean expression in response to GCV treatment, indicating that treated cells are arrested in the G1 phase before induction of apoptosis (Fig. 3d). High levels of P21 and arrest in G1 phase are also signs of myogenic differentiation, so we quantified gene expression for markers of myogenic differentiation: *MYOGENIN* to assess early stages of differentiation, *MYOMAKER* to evaluate cell fusion and *MYHC* to measure terminal differentiation. *MYOGENIN*, *MYOMAKER* and *MYHC* were all unchanged between treated and untreated cells (Fig. 3e). Thus, our proposed therapy causes a reduction in cell viability without induction of a more differentiated phenotype in remaining cells.

### RH30-derived mouse xenograft tumour size is reduced by the LV-miniMg-∆MEF3/NF1 suicide gene

**Fig. 4:**
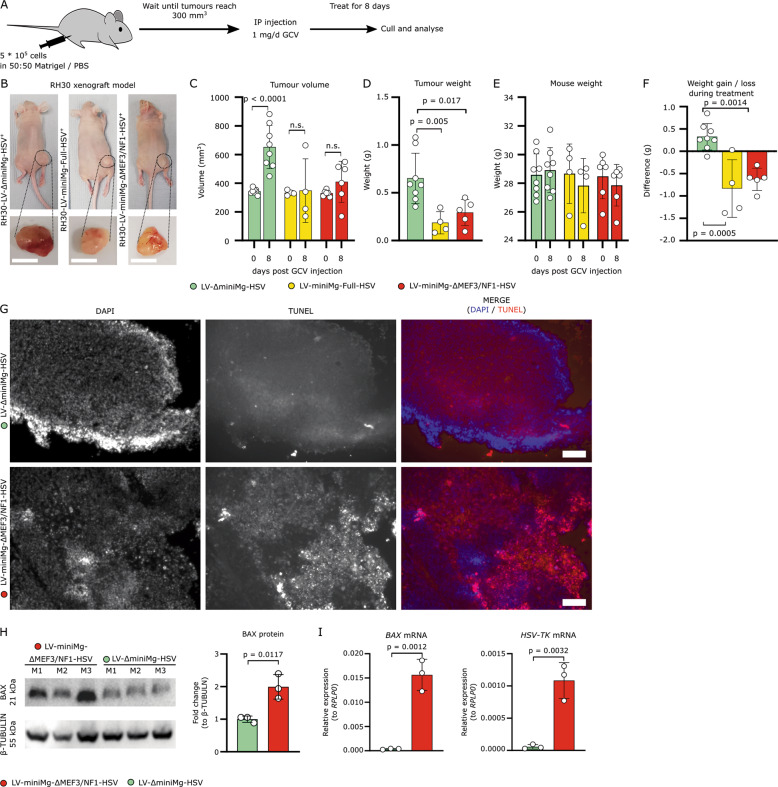
LV-miniMg-∆MEF3/NF1-HSV reduces ARMS tumour volume and weight via apoptosis in a xenograft mouse model. **a** Schematic depicting experimental regime where RH30 stably transduced with LV-∆miniMg-HSV, LV-miniMg-HSV or LV-miniMg-∆MEF3/NF1 were subcutaneously xenografted in the right flank of Swiss Nude mice to generate tumours. Once tumours reached 300 mm^3^, GCV was administered to the mice via IP injection for 8 days and tumours excised. **b** Representative images of mice just before tumours were excised and the tumours recovered. **c** Tumour volume measured with callipers a day before GCV administration and after 8 days of treatment. *N* = 4–8, statistical differences were assessed using a student’s *t*-test comparing values at days 0 and 10 in each group. **d** Excised xenografted tumour weight after 8 days of daily GCV administration. *N* = 4–8, significant differences were assessed using a one-way ANOVA with Dunnett’s post hoc test comparing each group to the control (LV-∆miniMg-HSV). **e** Mouse weighed a day before GCV administration and after 8 days of GCV treatment, *N* = 4–8 no significant differences between days 0 and 8 within each group detected with an unpaired student’s *t*-test. **f** Weight gain/loss of mice after 8 days of GCV administration, *N* = 4–8, significant difference was assessed with a One-Way ANOVA with Dunnett’s post hoc test comparing each group to the LV-∆miniMg-HSV control. All data are expressed as mean ± SD. **g** Representative images of TUNEL assays on tumour cryosections derived from LV-miniMg-∆MEF3/NF1-HSV and LV-∆miniMg-HSV samples. TUNEL^+^ cells are in red; nuclei are counterstained with DAPI. *N* = 3 mice per group. **h** Western blot for BAX and β-TUBULIN of tumour lysates from 3 LV-miniMg-∆MEF3/NF1-HSV mice and 3 LV-∆miniMg-HSV mice, with quantification of band intensity of BAX relative to β-TUBULIN, expressed as fold change of LV-miniMg-∆MEF3/NF1-HSV to LV-∆miniMg-HSV. Statistically significant differences were assessed with an unpaired student’s *t*-test. **i** RT-qPCR of *BAX* and *HSV-TK* mRNA expression in LV-miniMg-∆MEF3/NF1-HSV and LV-∆miniMg-HSV tumours relative to *RPLP0*. *N* = 3 for each group, statistical differences were assessed with an unpaired student’s *t*-test. Scale bar equals 1 cm (**b**) or 100 µm (**g**).

To evaluate the LV-miniMg-∆MEF3/NF1-HSV suicide gene in vivo we xenografted RH30 cells stably expressing LV-∆miniMg-HSV, LV-miniMg-Full-HSV or LV-miniMg-∆MEF3/NF1-HSV subcutaneously into the right flank of Swiss Nude mice. Once tumours reached ~300 mm^3^, the mouse was treated with daily intraperitoneal injections of 1 mg of GCV from the next day for 8 days and the tumour excised one day after the final GCV dose (day 9) and measured and weighed (Fig. 4a).

RH30 cells formed visible and palpable tumours in most mice, but over a variable timescale for all groups. At the end of the GCV treatment, tumour size was significantly increased in the control group (LV-∆miniMg-HSV), while there was no measurable difference in size in either LV-miniMg-Full-HSV or LV-miniMg-∆MEF3/NF1-HSV groups (Fig. 4b, c). The weight of excised tumours was also significantly reduced by LV-miniMg-Full-HSV or LV-miniMg-∆MEF3/NF1-HSV, compared to control LV-∆miniMg-HSV (Fig. 4d).

While the mean weight of mice in each group was unchanged during the administration of GCV (Fig. 4e), there was a significant difference in the mean change of weight during this period (Fig. 4f). Mice where the GVC-administration reduced mean tumour weight (LV-miniMg-Full-HSV and LV-miniMg-∆MEF3/NF1-HSV) also showed a net reduction in mean body weight during the treatment period, while control mice with LV-∆miniMg-HSV demonstrated a mean gain in body weight (Fig. 4f).

To confirm that apoptosis occurs at a higher rate in the LV-miniMg-∆MEF3/NF1-HSV sample group compared with the LV-∆miniMg-HSV control, we performed TUNEL staining on cryosections from excised tumours. Accumulation of DNA fragmentation was strongly visible in the LV-miniMg-∆MEF3/NF1-HSV group, while very little was detected in the LV-∆miniMg-HSV control group. TUNEL staining in the LV-miniMg-∆MEF3/NF1-HSV samples was often localized to specific areas (Fig. 4g and Supplementary Fig. 1).

To quantify apoptosis, we assessed BAX, an inducer of cytochrome C release-dependent apoptosis in response to severe DNA damage [35], a potential mechanism for HSV-TK induced apoptosis. Western blot for BAX from 6 independent tumour lysates revealed approximately twofold more BAX protein in LV-miniMg-∆MEF3/NF1-HSV tumours compared to LV-∆miniMg-HSV controls (Fig. 4h and Supplementary Fig. 1). RT-qPCR of mRNA isolated from six independent tumour lysates showed significantly higher expression of *BAX* too (Fig. 4i).

To investigate whether tumour cells were present after the 8 day treatment protocol that could still respond to GCV, we assayed *HSV-TK* expression, which was significantly increased in LV-miniMg-∆MEF3/NF1-HSV tumour samples compared with LV-∆miniMg-HSV controls (Fig. 4i). In summary, the LV-miniMg-∆MEF3/NF1 promoter can drive HSV-TK expression in vivo to slow/prevent tumour growth.

### The LV-miniMg-∆Mef3/NF1-HSV/GCV regime allows lowering of chemotherapy dose

Treating any solid tumour remains a challenge due to low drug penetrance and tumour cell heterogeneity that allows a population of resistant tumour cells to survive treatment and re-initiate tumour growth [36]. The HSV-TK/GCV system has the benefit of the bystander effect, where cells in close proximity to HSV-TK expressing cells are also targeted through the movement of phosphorylated GCV through gap junctions into non-expressing cells, so increasing effectiveness [37]. We tested the ability of our proposed suicide gene therapy to supplement the VAC (Vincristine/Actinomycin/Cyclophosphamide) chemotherapy combination commonly used in rhabdomyosarcoma treatment. RH30 cells expressing LV-miniMg-Full-HSV were treated with increasing concentrations of VAC and GCV to determine if there was an additive effect.

**Fig. 5:**
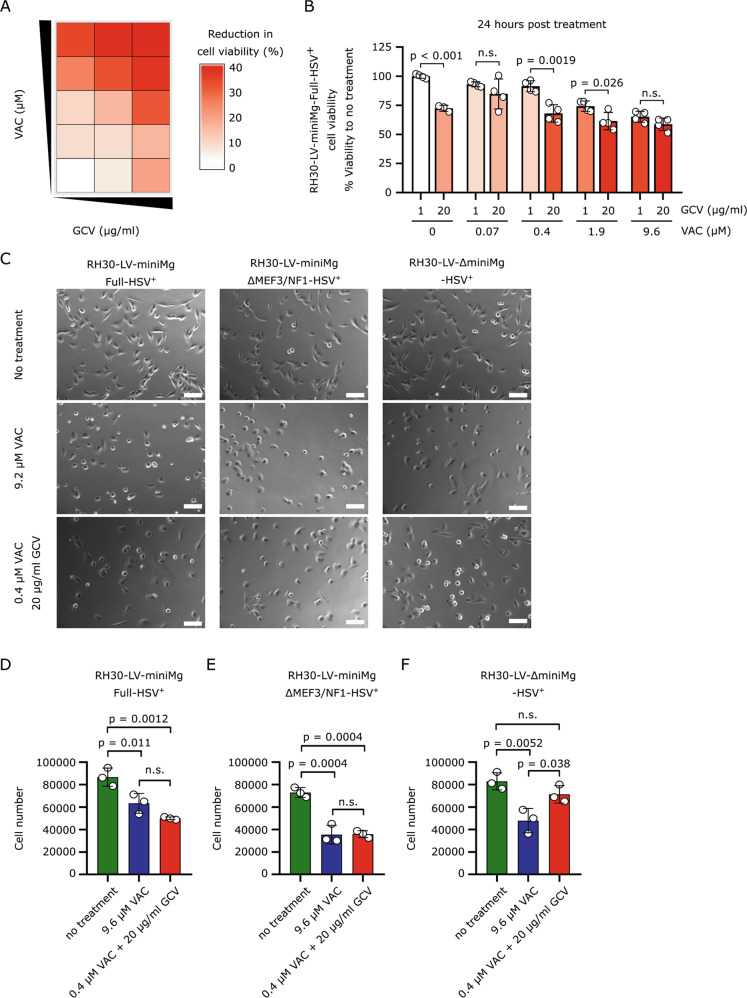
LV-miniMg-∆Mef3/NF1-HSV/GCV lowers the dose of chemotherapy. **a** Heatmap of dose-response of increasing VAC and GCV concentrations on LV-miniMg-Full-HSV-expressing RH30 cell viability, compared to no treatment (white block lower left). **b** Pair-wise comparison of the viability of RH30-LV-MiniMg-Full-HSV treated with either 1 or 20 µg/ml GCV and increasing concentrations of VAC. Significant differences between pairs was calculated using a student’s *t*-test, *N* = 3–4 replicates. **c** Representative brightfield images of RH30-LV-miniMg-Full-HSV^+^, RH30-LV-miniMg-∆MEF3/NF1-HSV^+^ and RH30-LV-∆miniMg-HSV^+^ without treatment or treated with 9.2 µM VAC or 0.4 µM VAC and 20 µg/ml GCV. **d**–**f** Mean cell number of RH30-LV-miniMg-Full-HSV^+^ (**d**), RH30-LV-miniMg-∆Mef3/NF1-HSV^+^ (**e**) and RH30-LV-∆miniMg-HSV^+^ (**f**) either untreated, treated with 9.2 µM VAC or 0.4 µM VAC and 20 µg/ml GCV. *N* = 3 biological replicates, statistical differences are calculated using a student’s *t*-test. All data are expressed as mean ± SD. Scale bar equals 100 µm.

After 24 h of treatment, viability of LV-miniMg-Full-HSV expressing RH30 cells was assessed and compared to low treatment control (0 µM VAC and 1 µg/ml GCV) (Fig. 5a). Cell viability was significantly decreased at nearly all concentrations of VAC (0, 0.4, 1.9 µM) if combined with 20 µg/ml GCV, compared to 1 µg/ml GCV (Fig. 5b). The decreased cell viability of LV-miniMg-Full-HSV expressing RH30 cells was identical when treated with either 9.2 µM VAC or with 0.4 µM VAC and 20 µg/ml GCV (34.8% decrease vs 31.9%).

We next compared 9.6 µM VAC with no treatment and the concentrations with the highest additive effect (0.4 µM VAC + 20 µg/ml GCV) on RH30 cells expressing *HSV-TK* under control of three different promoters. Quantification revealed that in LV-miniMg-Full-HSV or LV-miniMg-∆MEF3/NF1-HSV groups, RH30 cell numbers dropped significantly if cells were treated with 9.6 µM VAC or 0.4 µM VAC and 20 µg/ml GCV, compared to no treatment control (Fig. 5c–e). RH30 cells expressing *HSV-TK* under the LV-∆miniMg-HSV showed significantly reduced cell numbers after treatment with 9.6 µM VAC compared to the no treatment control, but treatment with 0.4 µM VAC and 20 µg/ml GCV had no effect (Fig. 5f). In conclusion, chemotherapy concentration can be lowered by a factor of 24 from 9.2 to 0.4 µM when supplemented with 20 µg/ml GCV in RH30 cells expressing LV-miniMg-Full-HSV or LV-miniMg-∆MEF3/NF1-HSV. Thus the concentration of chemotherapy can be significantly lowered to reduce the burden to the patient, if paired with our suicide gene therapy.

## Discussion

Treatment options and survival rates have recently increased for rhabdomyosarcoma, however survival rate for patients affected with the fusion-positive variant of ARMS is still <30% five years after diagnosis [38]. This highlights the need for novel therapeutic approaches.

To target ARMS tumour cells, the human minimal *MYOGENIN* promoter was selected, since *MYOGENIN* is constitutively expressed in rhabdomyosarcoma cells, but only during myogenic differentiation in skeletal muscle myoblasts, and not in other cell types. The minimal *MYOGENIN* promoter lacking both the NF1 and MEF3 binding motifs had enhanced specificity for ARMS over skeletal muscle myoblasts/myotubes.

The NF1 and MEF3 motifs are implicated in the activation of the skeletal muscle-specific human *aldolase A* alternative promoter pM [39]. The *NF1B* isoform is a putative downstream target of PAX3-FOXO1 [32], and we found that *NF1B* knockdown had opposite effects on MYOGENIN expression in C25 myoblasts and RH30 cells. While the role of NF1B in skeletal myogenesis or rhabdomyosarcoma is undescribed, it is highly expressed in multiple cancer types, including small cell lung cancer [40], melanoma [41], squamous cell carcinoma [42] and neuroendocrine carcinoma [43]. NF1B acts as a transcriptional activator or repressor, dependent on cellular context or regulatory region [44], and has divergent roles during the development, maintenance and differentiation of stem cells. It is not unsurprising then that it plays divergent roles in the regulation of gene expression in myoblasts versus RH30 cells.

The importance of the MEF3 motif in the regulation of *MYOGENIN* is well-described, as it is bound by SIX1 and SIX4, which together with MYOD, facilitate *MYOGENIN* expression during skeletal myogenesis [31]. Removal of the MEF3 site from the *MYOGENIN* reporter reduced reporter activity significantly in differentiating myoblasts. Interestingly removal of either the NF1B or MEF3 binding motif alone from the minimal *MYOGENIN* promoter was insufficient to abrogate HSV-TK activity in C25 human myoblasts, but the deletion of both motifs together enabled this. Effects could be indirect, as promoter shortening could reduce the accessibility of the DNA to other regulatory proteins. However, reduced toxicity in C25 myoblasts compared to RH30 cells suggests that regulation of this mutant human *MYOGENIN* promoter differs between cell types. Additional fine-tuning of the promoter sequence might reduce the low levels of activation in healthy myoblasts even further.

Forcible conversion of cells from proliferation to post-mitotic differentiation is a viable strategy for rhabdomyosarcoma treatment. As RH30 cells share characteristics with skeletal myoblasts, our suicide gene could also potentially induce differentiation, as well as apoptosis. However, differentiation markers including *MYOGENIN*, *MYOMAKER* and *MYHC* were unchanged in response to treatment of RH30-LV-miniMg-∆MEF3/NF1-HSV with GCV. Increased *CCND1* transcripts, possibly due to the accumulation of cells in G1, together with increased *P21* transcript, suggests that cells are inducing apoptosis. Together with the increase of Annexin 5 exposed on the cell surface, this strongly suggests that our suicide gene therapy is killing tumour cells.

Apoptosis was also detected in vivo after treating RH30-LV-miniMg-∆MEF3/NF1-HSV with GCV, confirming that it drives *HSV-TK* expression strongly enough, as also shown by the significantly smaller tumours observed. HSV-TK causes apoptosis through generating unrepairable DNA double-strand breaks [45], and in accordance with such DNA damage-induced apoptosis, a significant increase of BAX expression was observed in the LV-miniMg-∆MEF3/NF1 samples. *HSV-TK* was still strongly expressed after the treatment period, and it would be of interest to understand if that indicates a higher success is achievable with longer treatment, or that a resistant cell population remains after the initial treatment, as happens in response to various chemotherapeutic treatment regimens [46].

A major goal was to design a promoter specific for rhabdomyosarcoma cells over myogenic cells, and while we increased rhabdomyosarcoma specificity with our ∆MEF3/NF1 *MYOGENIN* promoter, there remains residual toxicity in myoblasts. Normally, a limitation of the HSV-TK1/GCV system is its ability to only affect dividing cells, but this is an advantage for muscle tissue, which is composed of mature, terminally differentiated post-mitotic skeletal muscle fibres, so refractory to the suicide gene. ARMS usually affects pediatric patients at an age when satellite cell-derived myoblasts will be required for muscle fibre growth/hypertrophy. The minimal activity of our mutant human *MYOGENIN* promoter in myoblasts may be a concern, but any corresponding toxic effect should only be exerted on myoblasts as they enter myogenic differentiation, leaving mature muscle unaffected. Chemo/radiotherapy also affects dividing cells though, but are used in children/adolescents where there are no alternatives. Targeting the therapy to tumours and adjusting dosing regimens could mitigate off-target effects on skeletal muscle.

Both viral and non-viral targeting options exist for gene delivery. Liposomal nanoparticles can deliver SiRNA against PAX3-FOXO1 efficiently into in vivo ARMS models, delaying tumour initiation/growth, but failed to induce apoptosis [47]. Targeting peptides have been developed that show specificity to RH30 [48], and these could modify liposomal carriers to enhance specificity. Among other factors, effective delivery of plasmid DNA into the nucleus is dependent on the size of the pDNA. We could reduce size by removing LV sequences to only retain the modified *MYOGENIN* promoter driving HSV-TK. An NLS could further enhance nuclear import [49]. Adeno-associated viruses (AAVs) with high tropism for muscle such as AAV2 or AAV9 could also be promising candidates for delivery. Inclusion of a tumour targeting sequence (e.g. NGRAHA, containing the NGR motif facilitating integrin binding) increases uptake by RD cells 10–20× compared to wildtype AAV2 [50]. Tumour cell selective killing by HSV-TK delivered by an AAV was successful for hepatocellular carcinoma [51] and oral squamous cell carcinoma [52] in mice, suggesting that AAV delivery is viable.

Another general challenge with cancer treatment is the low drug penetrance of tumour tissue [53]. The HSV-TK/GCV system benefits from a strong bystander effect, mediated through GAP-junctions of tightly connected cells, which extends treatment beyond primary transduced cells. HSV-TK shows a strong affinity towards thymidine though, requiring high concentrations of GCV to be administered. This may explain why we required a GCV concentration of ≥10 µg/ml to induce apoptosis, in the upper range used in vitro. Higher affinity substrates exist for HSV-TK, such as acyclovir [54]. Furthermore, HSV-TK efficiency differs between cell types [55], and HSV-TK mutants show varying efficiencies towards their substrate [56]. Fine-tuning HSV-TK variant and substrate should generate a combination to reduce potential side-effects of the HSV-TK/GCV system.

It is unlikely that any therapy could kill 100% of tumour cells, even with full tumour penetrance, as intra-tumoral heterogeneity often renders sub-populations of cells resistant. However, synergistic effects of treatments with HSV-TK/GCV occurs in murine and human colon carcinoma cells [57], where survival of nude mice was higher if GCV was used with the topoisomerase inhibitor topotecan. In addition, HSV-TK/GCV suppresses cell growth in chemo resistant K562 and THP-1 cells (leukaemia) [58]. Evaluating our suicide gene therapy with a standard chemotherapy regime (VAC) showed identical effects even if we reduced the chemotherapy concentration by >24× from 9.6 to 0.4 µM if supplemented with 20 µg/ml GCV.

In conclusion, we generated a modified human *MYOGENIN* promoter by deleting MEF3 and NF1 binding sites that has enhanced specificity for rhabdomyosarcoma over skeletal myogenic cells. Our LV-miniMg-∆MEF3/NF1 promoter is capable of driving *HSV-TK* to levels sufficient to induce apoptosis in ARMS cells, but not myoblasts. Such a LV-miniMg-∆MEF3/NF1 directed suicide gene therapy could also lower doses of chemotherapeutic agents if used in combination.

## Supplementary information

Supplemental Figure
