# Correction: A human *Myogenin* promoter modified to be highly active in alveolar rhabdomyosarcoma drives an effective suicide gene therapy

**DOI:** 10.1038/s41417-020-00251-y

**Published:** 2020-11-11

**Authors:** Johanna Pruller, Isabella Hofer, Massimo Ganassi, Philipp Heher, Michelle T. Ma, Peter S. Zammit

**Affiliations:** 1 King’s College London, Randall Centre for Cell and Molecular Biophysics, London, SE1 1UL UK; 2 King’s College London, School of Biomedical Engineering and Imaging Sciences, St Thomas’ Hospital, London, SE1 7EH UK

**Keywords:** Gene regulation, Targeted therapies, Sarcoma

Correction to: *Cancer Gene Therapy*

10.1038/s41417-020-00225-0 published online 25 September 2020

This Article was originally published with an incorrect title: “A modified human *Myogenin* promoter that is highly active in alveolar rhabdomyosarcoma” instead of “A human *Myogenin* promoter modified to be highly active in alveolar rhabdomyosarcoma drives an effective suicide gene therapy”. This has now been corrected in both the PDF and HTML versions of the Article.

