# Supplementary material for: A human Myogenin promoter modified to be highly active in alveolar rhabdomyosarcoma drives an effective suicide gene therapy
Source: Cancer Gene Ther. 2020 Sep 25;28(5):427–41. doi: 10.1038/s41417-020-00225-0 (PMC8119243; doi:10.1038/s41417-020-00225-0)
Supplement: Supplementary file 1 — Supplemental Figure [file 41417_2020_225_MOESM1_ESM.pdf]

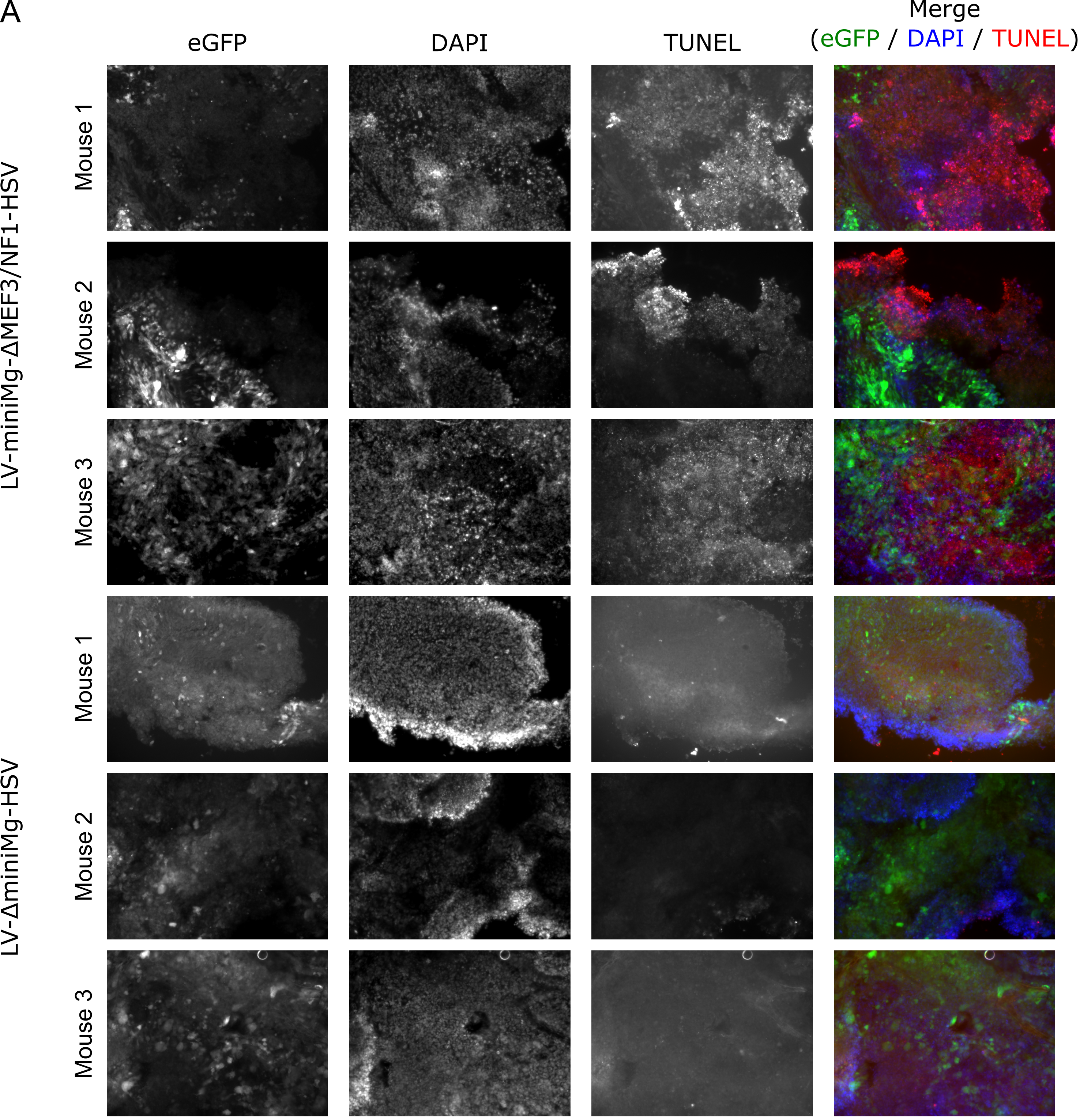

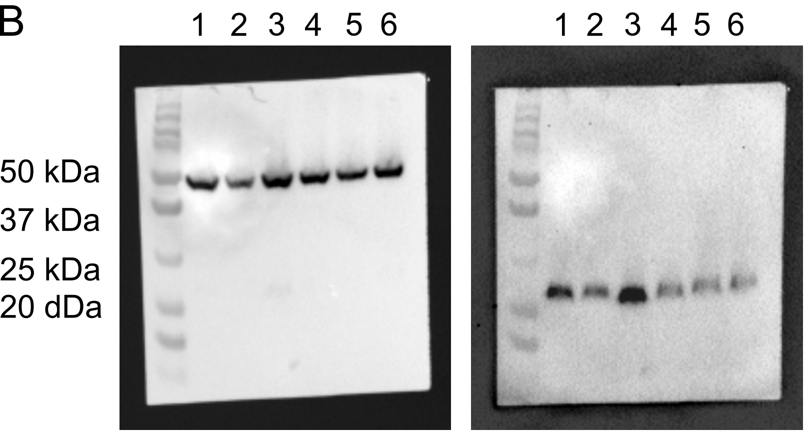

Samples 1 - 3: LV-miniMg-ΔMEF3/NF1-HSV, Mouse 1 - 3

Samples 4 - 6: LV-ΔminiMg-HSV, Mouse 1 - 3

Supplementary Figure 1: (A) TUNEL assay of tumour derived sections of 3 mice of the LV-ΔminiMg-HSV and LV-miniMg-ΔMEF3/NF1-HSV groups. TUNEL+ cells are in red; nuclei are counterstained with DAPI . (B) Full membrane images of western blot , detecting BAX and β - TUBULIN in tumour lysates of 3 mi ce of the LV-ΔminiMg-HSV group and the LV-miniMg-ΔMEF3/NF1-HSV group.
